# Dental Education

**Published:** 1875-06

**Authors:** 


### EDITORIAL, ETC.

*Dental Education.*—At the present time considerable discussion is going on in the different dental societies and journals concerning this subject, some writers and speakers criticising and others defending the colleges in their efforts to advance the proficiency of those who resort to these institutions for the purpose of laying the foundation for the successful and scientific pursuit of their chosen profession. That there is a great contrast between the members of the medical and dental professions in this respect, must be apparent to all familiar with the subject of medical and dental education.

While it is rare to find an article in a medical journal, or a speaker at a medical association meeting condemning their own Colleges, or in any way criticising the course of study, except to recommend longer sessions, the case is altogether different with the dental profession. At every dental association meeting and in some of the journals. every possible argument is brought forth to oppose the Colleges and injure the men who for years have been expending labor, and sacrificing time and means in advancing the standard of dental education.

It is satisfactory, however, to know that the great majority of these critics have not been within the walls of any of these institutions for years, and are wholly ignorant of the progress and working of all the old and well established dental colleges, and hence are far from being competent judges. Besides, there are to be found in every community persons who find it much easier to censure than to correct, and also those who imagine that there can be no good in any institution in which they have not a controlling power, or one conducted otherwise than according to their own peculiar views. We venture the assertion and truly believe it to be a correct one, without desiring to make any invidious comparison, that the requirements of all the old dental colleges of this country, are at the present time far beyond what will be found in any medical college, and that more candidates for graduation are rejected, in proportion to the size of the classes, in the dental than in the medical colleges.

We may refer for example, to the late session of the Baltimore College of Dental Surgery, when nearly *one third* of the whole number who composed the senior or graduating class failed to receive the degree of Doctor of Dental Surgery at the final examination. What medical college has rejected so many as unqualified? And yet there are members of the dental profession who either through ignorance or wilfulness continue to harp upon this subject, and cry out that the colleges are not advancing with the demands of the science.

It would be much more to the credit and reputation of such persons if they would imitate the example of their medical brethren and endeavor to support the colleges, being justified in this by visiting these institutions and becoming enlightened as to their doings and workings.

We are satisfied that the Faculties of all the well established dental colleges in this conntry are doing all in their power to advance dental education, and that the course of study in all of these old institutions is far superior to what it was ten or even five years ago.

We are pleased to see that at a late meeting of the Pennsylvania Odontographic Society, the proceedings of which are published in the *Pennsylvania Journal of Dental Science*, the colleges had some faithful and well informed advocates, as the following extracts will show:

Dr. Allen said that when he hears that dental education has been a signal failure, it carries him back to the time when we were destitute of the splendid advantages we now have. Within the last thirty years there has been as much advance made in dentistry, and perhaps more, than in any other profession. The agitation of this question reminds me of the famous saying of Ajax: "Give me light; 'tis all that Ajax wants."

Dental Colleges are the true exponents of this subject. The system of practical education as well as theoretical, is the only way to establish a true system of dental education. The hands and fingers in dentistry must be educated as well as the mind.

It is therefore best for a student to avail himself of every kind of learning that will have a bearing upon the subject. After all, there is much to be learned by intuition ; let the mind be turned inward. The man that thinks he is perfect because he has learned all that the professors can teach him, is a hopeless case. In the best and most hopeful cases mistakes are made by young men in dentistry as well as in medicine.

We should not be divided into specialties. It is an error to have mechanical dentists and operative dentists. The whole field should be taken in, and to meet the wants of the age a well educated dentist must be prepared to take on any case for treatment that may present itself. The result of a division would be to run either branch into mechanical manipulation as the leading qualification, and then instead of a higher standard being followed, the country would be flooded with the worst kind of work. It must be admitted that the mechanical branch is of as much importance as the operative, and that to do such work properly, a thorough study of the action of the muscles of the mouth and face and the part they take in the formation of the contour of the face should be thoroughly understood.

We are called upon to restore lost or absorbed substance in order to reproduce a natural expression. This needs mechanical ingenuity, and theoretical knowledge. It needs an education that calls into action all that is taught in the colleges.

J. Foster Flagg, D. D. S., said thirty odd years ago Hayden, Harris, Parmlee and others were interested in the status of dentistry, as we are to-day. when they started a society and a college to establish a profession. But when an effort was made to obtain medical recognition, they got the cold shoulder. It has been a bloody fight ever since, and those who are ignorant of its progress, are perhaps, the most eager to criticise.

As to a phillippic upon the medical profession, I do not now desire to take up any of your time. I am here to tell you that dentistry as it was started then, has become a live profession, and that the flag is still floating. All I know, and all I want to know about medicine, is that which belongs to, and is taught in the dental Colleges. If a dental education is desired, it must be attained at dental Colleges and nowhere else. It is a preposterous idea to call dentistry into question as a profession.

Though it might not, be as old, it has done as much for science as medicine.

We gave them nitrous oxide and ether, and they gave us chloroform. Dentistry has grown marvelously and is a profession in its literature, its societies and its colleges. You do not find an analogous case in any specialty. It contains all the resources of a living, energetic profession.

Dentistry is enough for me. I am satisfied to be called a dentist. The D. D. S. satisfies my ambition, but the D. M. D. is no title at all; it stands for deficient medical doctor. If the D. D. S. flies, I fly, and if it dies, I die.

Prof. J. H. McQuillen said we live in an age where reform and progress are earnest and active on every side; and to make those elements the more effectual, criticism should be invited, and fairly and truthfully exercised. In the history of dentistry it will be seen that many have entered its ranks from other occupations. Some, who were men of culture and energy, and seeing that dentistry was more than a mere handicraft, exerted their energies and intelligence to raise it to the status of a first-class profession. Step by step the work of improvement was led forward. First a magazine, then an association, and then a college. Hayden, Harris and others made for themselves enviable and imperishable reputations in this work of building up a cultus and a science.

After the establishment of the Baltimore College, delegates were duly appointed, who sought admission as representatives in a leading medical association, but were rejected. A proposition was made by Dr. Gardet to establish a dental professorship in a medical college, but all was rejected on the ground of dentistry being a mere handicraft, Thus to the minds of those positive, progressive men, the establishment of a dental school became a necessity.

What to-day is the course of instruction in the dental colleges? Anatomy, physiology, pathology and therapeutics are taught to the same extent as in medical colleges. At the same time there are established departments, where mechanical manipulation and operative dentistry are taught. Clinical operations are also brought in, and ample instruction given. The student can thus carry out that which he has seen performed by skilled and experienced men. They have ample opportunity to put into practice at the colleges what they learn from the above sources; and if they fail, the demonstrators are there to instruct and correct. Thus the science and art of dentistry is not only alone taught, but the practice also. This cannot be acquired in two four or six years in medical schools. There the mind is crowded by a multiplicity of studies and lectures, and there is no opportunity of acquiring a practical knowledge of the profession. Hence, when a student goes out into the world he is without practical knowledge, and must gain it from experiments upon the theory he may possess, when often the gravest mistakes occur.

There is here no desire to make invidious comparisons between medical and dental colleges. But in their own peculiar spheres the dental colleges have in this particular a signal advantage. The more, therefore, the subject is agitated the more these important advantages in dental education, as it is developed in our schools, are brought to light and they reap the advantage, for they seem to flourish better and are held in higher repute by the masses. Those who criticise with so little knowledge, as they seem to possess of the workings and doings of dental colleges, might profit by devoting some to themselves.

